# Supplementary material for: The Progression of Symptoms in Post COVID-19 Patients: A Multicentre, Prospective, Observational Cohort Study
Source: Biomedicines. 2024 Oct 30;12(11):2493. doi: 10.3390/biomedicines12112493 (PMC11591596; doi:10.3390/biomedicines12112493)
Supplement: Supplementary file 1 [file biomedicines-12-02493-s001.zip › Supplementary Material/Figure S1.pdf]

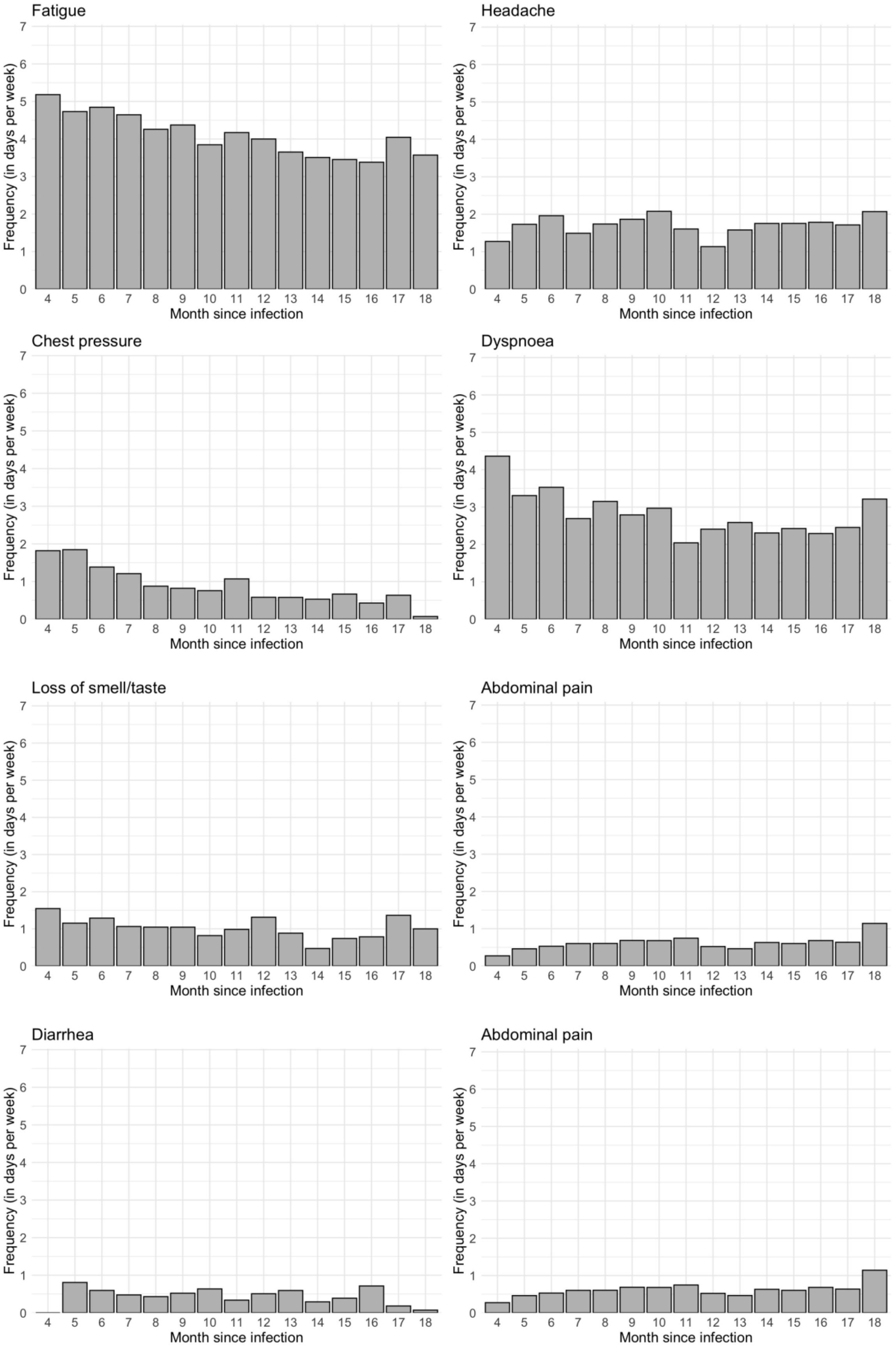

Figure S1. Mean weekly frequency per symptom per month. Amount of questionnaires per month: 4 (n=11), 5 (n=26), 6 (n=56), 7 (n=64), 8 (n=66), 9 (n=67), 10 (n=66), 11 (n=71), 12 (n=67), 13 (n=69), 14 (n=66), 15 (n=54), 16 (n=42), 17 (n=22), 18 (n=14)
