# Supplementary material for: The Progression of Symptoms in Post COVID-19 Patients: A Multicentre, Prospective, Observational Cohort Study
Source: Biomedicines. 2024 Oct 30;12(11):2493. doi: 10.3390/biomedicines12112493 (PMC11591596; doi:10.3390/biomedicines12112493)
Supplement: Supplementary file 1 [file biomedicines-12-02493-s001.zip › Supplementary Material/Questionnaire.pdf]

Questionnaire on disease progression and adverse events

Date: ________

1. Did you visit your general practitioner in the past month?
    - ☐ Yes
    - 1.1 Have you been newly diagnosed at that time?
        - ☐ Yes, diagnosis: ____
        - ☐ No
    - ☐ No

2. Did you visit a specialist at the hospital in the past month?
    - ☐ Yes
    - 2.1 Have you been newly diagnosed at that time?
        - ☐ Yes, diagnosis: ____
        - ☐ No
    - ☐ No

3. Did you experience the following symptoms in the past two weeks?

3.1 Fatigue
- ☐ Yes
- 3.1.1 How often?
    - ☐ Weekly, how often per week: ________
    - ☐ Daily, how often per day: ________
- ☐ No

3.2 Headache
- ☐ Yes
- 3.2.1 How often?
    - ☐ Weekly, how often per week: ________
    - ☐ Daily, how often per day: ________
- ☐ No

3.3 Chest pressure
- ☐ Yes
- 3.3.1 How often?
    - ☐ Weekly, how often per week: ________
    - ☐ Daily, how often per day: ________
- ☐ No

3.4 Dyspnoea
- ☐ Yes
- 3.4.1 How often?
    - ☐ Weekly, how often per week: ________
    - ☐ Daily, how often per day: ________
- ☐ No

3.5 Loss of smell and/or taste
- ☐ Yes
- 3.5.1 How often?
    - ☐ Weekly, how often per week: ________
    - ☐ Daily, how often per day: ________
- ☐ No

3.6 Abdominal pain

- ☐ Yes
- 3.6.1 How often?
  - ☐ Weekly, how often per week: _______
  - ☐ Daily, how often per day: _______
- ☐ No

3.7 Diarrhoea

- ☐ Yes
- 3.7.1 How often?
  - ☐ Weekly, how often per week: _______
  - ☐ Daily, how often per day: _______
- ☐ No

3.8 Obstipation

- ☐ Yes
- 3.8.1 How often?
  - ☐ Weekly, how often per week: _______
  - ☐ Daily, how often per day: _______
- ☐ No

3.9 Other

- ☐ Yes, which symptom and how often? ____
- ☐ No

4. Are you using medication?
   - ☐ Yes
   - 4.1 Is there any change in your medication use?
     - ☐ Yes, specify: ____
     - ☐ No
   - ☐ No
